# Editorial: Molecular Vaccines Against Pathogens in the Post-genomic Era

**DOI:** 10.3389/fcimb.2019.00443

**Published:** 2020-01-22

**Authors:** Angel Alejandro Oñate, Yanmin Wan, Alberto Moreno

**Affiliations:** ^1^Laboratory of Molecular Immunology, Department of Microbiology, Universidad de Concepción, Concepción, Chile; ^2^Department of Infectious Disease, Huashan Hospital, Fudan University, Shanghai, China; ^3^Emory Vaccine Center, Yerkes National Primate Research Center, Emory University, Atlanta, GA, United States; ^4^Division of Infectious Diseases, Department of Medicine, Emory University, Atlanta, GA, United States

**Keywords:** vaccines, systems biology, multi-omics, immunoinformatics, modern vaccinology

The development and implementation of vaccines are one of the greatest Public Health achievements in human history (Centers-for-Disease-Control-and-Prevention, 2011a,b). In the first decade of the twenty-first century, the Expanded Program on Immunizations averts more than 2.5 million deaths every year (WHO, 2009). Vaccines not only prevent deaths, disease, and disability but also provide community protection by reducing the spread of the disease within a population (Orenstein and Ahmed, 2017). In the US alone, it has been estimated that the prevention of clinical cases and deaths by vaccination for a single birth cohort represents a net savings of $68.8 billion in total societal costs (Zhou et al., 2014). Although there are vaccines internationally available against 26 infectious diseases, nearly half of all deaths from infectious diseases are caused by pathogens for which no vaccine is available (Piot et al., 2019), including emerging and re-emerging pathogens (Williamson and Westlake, 2019). Interestingly, the majority of these vaccines have been developed empirically with limited information available on the mechanisms involved in protection (Pulendran and Ahmed, 2011). The development of high-throughput technologies and the advances in bioinformatics allow the massive generation and integration of datasets from multiple components of a biological system to understand in-depth physiological or pathological events (Pezeshki et al., 2019). This holistic approach of systems biology, when applied to studies of vaccine-induced immune responses, is known as system vaccinology (Pulendran et al., 2010). This research field will provide tools not only for the rational vaccine design but also for the development of novel adjuvants and vaccine delivery systems (Raeven et al., 2019). In this *Frontiers* Research Topic, some concepts of modern vaccinology are explored.

## Influenza Pandemic Preparedness

In light of the new technological advances in systems biology, Short et al. reviewed host, pathogen, and environmental factors that determined the high mobility and mortality rates reported for the 1918 influenza pandemic. The progress in understanding the immune responses induced by influenza vaccines using system biology tools is reviewed by Sherman et al. The authors summarized the molecular signatures of B cell responses that have been reported to correlate with the response to vaccination. Signatures of long-lasting antibody responses and immunosenescence defined after influenza vaccination are also discussed. The availability of influenza challenge models combined with system biology tools has started to reveal differences in gene expression signatures in volunteers exposed either to H1N1 or H3N2. Furthermore, access to human challenge models has also shown the dynamics of the viral evolution within the host. Jang and Seong review recent data on the advances and challenges on the development of universal influenza vaccines (UIVs). The search for a broadly protective vaccine capable of redirecting the immune responses from the variable immunodominant regions to conserved subdominant has identified potential candidates that can be delivered using novel vaccine platforms. Among the strategies, novel immunoinformatics tools to define consensus or ancestral sequences by phylogenetic analyses are discussed. Systems vaccinology approaches are needed to understand the mechanism of protections induced by UIVs.

## Interactome Mining to Reveal Pathogen Targets

Characterizing the patterns of molecular interaction of proteins, using protein interaction networks or interactomes, is essential for understanding the cellular function and pathogen–host interactions. Mujawar et al. report the development of protein interactomes for *Acinetobacter baumannii*, a causative agent of nosocomial infections, to identify potential vaccine candidates and virulence factors for immunological or pharmacological targeting. The identified proteins are then mapped onto the whole genome protein interactome for *in silico* verification to generate a short list of proteins for future *in vivo* validation.

## Adjuvant Formulations

The development of vaccines based on subunits is a promising strategy given its excellent safety profile. However, candidates are usually poorly immunogenic and require the formulation with potent adjuvants. In their article, Contreras et al. present new data on tick vaccination, using a combination of the recombinant subolesin from *Rhipicephalus microplus* described as protective for this arthropod in combination with heat-inactivated *Mycobacterium bovis* and administered orally. The results of this study confirmed the efficacy of subolesin-based vaccines for the control of cattle tick infestations and expanded to oral vaccination using an immunostimulant. Ebensen et al. report the use of a combination of c-di-AMP, a STING agonist, and a promising adjuvant capable of stimulating an effective Th1/Th2 and cytotoxic immune response, with the well-known adjuvant alum. This adjuvant system was tested with a model antigen showing the induction of a balanced humoral and cellular immune responses.

## Delivery Systems

In the search for novel vaccine delivery systems, several strategies have been proposed. Zurita et al. address the need to develop more effective acellular pertussis vaccines. In eliciting tissue-resident memory CD4+ T cells, a critical effector subset involved in protection, the authors show that an outer membrane vesicle (OMV)-based vaccine is more effective than a commercial acellular vaccine, having a better ability to induce protection and immunological memory. Chimeric virus-like particles (VLPs) using the murine polyomavirus are reported to be an effective platform to deliver subunit vaccines by Pattinson et al. In proof-of-concept studies, the authors produced chimeric VLPs genetically modified for surface expression of CD8+, CD4+, or B cell epitopes derived from the *Plasmodium yoelii* circumsporozoite protein. The vaccine platform was efficient to induce CD8+ T cell and antibody responses, but limited CD4+ T cell responses. The potential of using Porcine Circovirus Type 2 (PCV2) Chimeric VLPs for surface expression of exogenous peptides is presented by Wang et al.. The availability of three-dimensional structural data of the PCV2 capsid protein allows the authors to use homology modeling to characterize surface displaying. The platform allows the insertion of foreign peptides without altering the virus assembly and its entrance to the host cell.

DNA vaccines consist of plasmid vectors that, after immunization, allows intracellular expression of the encoded antigens. Protective efficacy is achieved by the induction of a strong humoral and cellular immune response dependent on B and T cells. In a mini review by Mekonnen et al., the limitations and strategies for using DNA vaccines against human immunodeficiency virus (HIV)-1 and hepatitis C virus (HCV) in humans are discussed. The authors review the potential of DNA vectors for elicit protective compartmentalized CD8 + T cells in the liver for HCV and the genito-rectal mucosa for HIV.

Conjugation of poorly immunogenic antigens to carrier protein is the strategy used for glycoconjugate vaccines. McCaffery et al. reported the use of genetic conjugation to deliver a *Plasmodium vivax* sexual-stage vaccine candidate. The authors take advantage of a highly immunogenic chimeric protein that they designed targeting a blood-stage antigen to create a bifunctional vaccine by genetic linkage to the transmission-blocking vaccine candidate Pvs25. This approach addresses the need for the development of effective multi-stage malaria vaccines.

## Closing Perspectives

The wealth of information provided by systems biology approaches can be integrated into product design for the development of novel vaccines. This Research Topic offered a glimpse into some strategies in modern vaccinology. Although these tools are essential for the development of effective vaccines against agents with complex host–pathogen interactions such as HIV, tuberculosis, and malaria, from the global health perspective, it is also critical to identify and deal with factors associated with persistent social disparities. It has been estimated that only 5% of all children born worldwide receive all 11 vaccines recommended by the WHO (Mantovani and Santoni, 2018). It is therefore critical that in conjunction with research efforts to develop novel vaccines, global health initiatives such as the Global Alliance for Vaccines and Immunizations (GAVI) that promotes equal access to vaccines are strengthened (Ikilezi et al., 2019; Rappuoli et al., 2019; Zerhouni, 2019).

## Author Contributions

All authors listed have made a substantial, direct and intellectual contribution to the work, and approved it for publication.

### Conflict of Interest

The authors declare that the research was conducted in the absence of any commercial or financial relationships that could be construed as a potential conflict of interest.
